# Facile Combination of Bismuth Vanadate with Nickel Tellurium Oxide for Efficient Photoelectrochemical Catalysis of Water Oxidation Reactions

**DOI:** 10.1021/acsami.4c07117

**Published:** 2024-09-05

**Authors:** Yu-Hsuan Chiu, Ren-Jei Chung, Chutima Kongvarhodom, Muhammad Saukani, Sibidou Yougbaré, Hung-Ming Chen, Yung-Fu Wu, Lu-Yin Lin

**Affiliations:** † Department of Chemical Engineering and Biotechnology, National Taipei University of Technology, Taipei 10608, Taiwan; ‡ Department of Chemical Engineering, King Mongkut’s University of Technology Thonburi, 126 Pracha-u-thit, Toong-kru, Bangkok 10140, Thailand; § Department of Chemical Engineering, University of New Brunswick, Fredericton, New Brunswick E3B5A3, Canada; ∥ Department of Mechanical Engineering, Faculty of Engineering, Universitas Islam Kalimantan MAB, Jl. Adhyaksa No. 2, Banjarmasin 70124, Indonesia; ⊥ Institut de Recherche en Sciences de la Santé (IRSS-DRCO)/Nanoro, Ouagadougou 03 7192-03, Burkina Faso; # Gingen Technology Co., LTD., Rm. 7, 10F., No. 189, Sec. 2, Keelung Road, Xinyi District, Taipei 11054, Taiwan; ¶ Department of Chemical Engineering, Ming Chi University of Technology, New Taipei City 24301, Taiwan

**Keywords:** bismuth vanadate, cocatalyst, light absorbance, nickel tellurium oxide, surface engineering, water oxidation

## Abstract

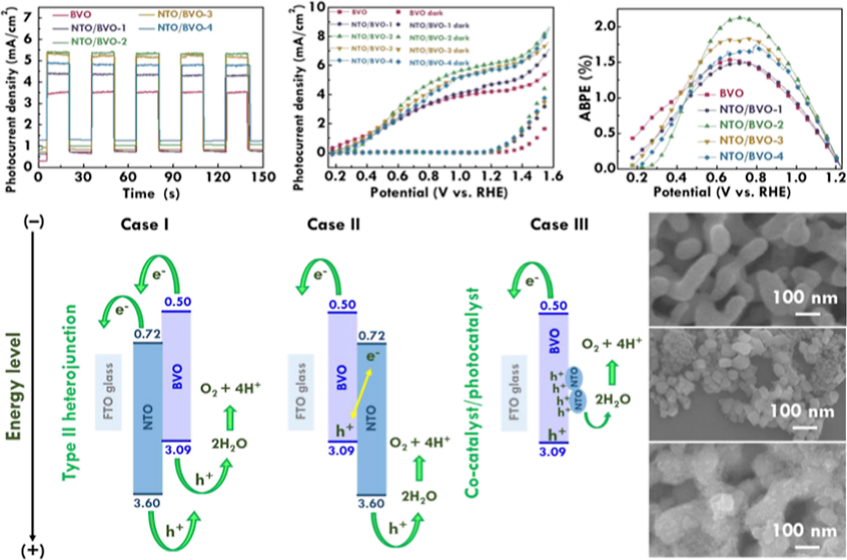

Bismuth vanadate (BVO) having suitable band edges is one of the effective photocatalysts for water oxidation, which is the rate-determining step in the water splitting process. Incorporating cocatalysts can reduce activation energy, create hole sinks, and improve photocatalytic ability of BVO. In this work, the visible light active nickel tellurium oxide (NTO) is used as the cocatalyst on the BVO photoanode to improve photocatalytic properties. Different NTO amounts are deposited on the BVO to balance optical and electrical contributions. Higher visible light absorbance and effective charge cascades are developed in the NTO and BVO composite (NTO/BVO). The highest photocurrent density of 6.05 mA/cm^2^ at 1.23 V versus reversible hydrogen electrode (V_RHE_) and the largest applied bias photon-to-current efficiency (ABPE) of 2.13% are achieved for NTO/BVO, while BVO shows a photocurrent density of 4.19 mA/cm^2^ at 1.23 V_RHE_ and ABPE of 1.54%. Excellent long-term stability under light illumination is obtained for NTO/BVO with photocurrent retention of 91.31% after 10,000 s. The photoelectrochemical catalytic mechanism of NTO/BVO is also proposed based on measured band structures and possible interactions between NTO and BVO. This work has depicted a novel cocatalytic BVO system with a new photocharging material and successfully achieves high photocurrent densities for catalyzing water oxidation.

## 1 Introduction

To solve the fossil fuel pollution and energy shortage problems, generating clean and sustainable energy is of great significance. Hydrogen is regarded as the most efficient chemical fuel with great energy density.^1−3^ Several methods have been proposed to produce hydrogen, such as steam reformation and desulfurization.^4,5^ However, these commonly applied hydrogen production methods may produce a lot of pollution. The photoelectrochemical (PEC) water splitting is a relatively new hydrogen production method, which requires merely electricity or light for driving and produces almost no pollution.^6−8^ The PEC water splitting system is composed of a photoanode and a photocathode, which are, respectively, responsible for the photocatalytic water oxidation and reduction. The oxygen evolution reaction (OER) happening in the PEC system requires a four-electron transfer path. Therefore, OER is more sluggish and requires effective electro/photocatalysts for initiating.^9,10^ Till now, there is still a challenge to design low-cost, highly stable, and catalytically efficient photocatalysts for driving the OER and realize effective water splitting in the PEC system.

Bismuth vanadate (BVO) is one of the effective photocatalysts for the OER, owing to the small band gap and suitable band edges. Nevertheless, sluggish surface/bulk charge separations and weak photon responses in the partial visible-light region limit the photocatalytic performance of BVO.^11−18^ Researchers have been devoted to creating various strategies to extend the visible-light absorbance and charge-transfer efficiency in the photocatalyst or photoelectrocatalyst systems, such as designs of preferable morphology,^19−21^ doping of heteroatoms,^11,22^ constructions of heterojunction,^23−26^ or incorporations of cocatalysts.^27−32^ Chen and Lin fabricated cone-shaped BiVO_4_ nanorod arrays on the conductive substrate via a solution process for photoelectrochemical catalytic water oxidation applications. A photocurrent density of 1.00 mA/cm^2^ at 1.23 V_RHE_ was obtained due to high crystallinity, preferable (040) crystal plane, small charge-transfer resistances, and high carrier density.^16^ Xiao et al. synthesized the BiVO_4_ nanorod array on FTO glasses via a simple solution method and applied as the photoanode for water oxidation. A photocurrent density of 0.12 mA/cm^2^ at 1.23 V_RHE_ and an onset potential of 0.32 V_RHE_ were obtained.^33^ Ma and co-workers proposed a solid-state synthesis to fabricate BiVO_4_ by mixing bismuth and vanadium salts without other mediums and directly annealing the mixture at 450 °C. A photocurrent density of 0.21 mA/cm^2^ at 1.23 V_RHE_ and an onset potential of 0.686 V_RHE_ were obtained.^11^ Chen et al. propose the synthesis of bismuth sulfide/BVO nanorod array on conducting glasses via solution and hydrothermal reactions to construct a type II heterojunction. A higher photocurrent density of 1.43 mA/cm^2^ were obtained for bismuth sulfide/BVO electrode than that for the BVO electrode (0.25 mA/cm^2^) at 1.23 V_RHE_.^26^ Liu et al. developed vanadium oxide with enriched oxygen vacancies as the oxygen evolution cocatalyst for BVO photoanodes. A photocurrent density of 6.29 mA/cm^2^ at 1.23 V_RHE_ and a charge-transfer efficiency of 96% were attained.^34^ Among all, decorating cocatalysts is considered as a facile way to significantly enhance the photoelectrocatalytic ability of BVO since the intrinsic properties of BVO can possibly maintain, and the cocatalyst can be synthesized separately. Numerous cocatalysts have been proposed to decorate BVO for establishing the cocatalyst/photocatalyst systems. Qi et al. demonstrated enhanced OER on BVO via facet-selective photodeposition of FeCoO_*x*_ as the dual-cocatalyst. The resulting PEC water splitting system shows the solar-to-hydrogen conversion efficiency of 12.3%.^27^ Wu and co-authors introduced W-doped BVO electrodes combined with the cocatalyst of NiO_*x*_(OH)_*y*_ as a photoanode material for the PEC oxidation of glycerol.^28^ Pilli et al. electrochemically deposited a cobalt phosphate-based OER cocatalyst onto Mo-doped BVO. A photocurrent density of 1.0 mA/cm^2^ at 1.0 V_Ag/AgCl_ was achieved in 0.5 M Na_2_SO_4_.^29^ Cheng and co-workers decorated oxidized ZIF67 as a cocatalyst on W-doped BVO using the drop casting technique for catalyzing photoelectrochemical water oxidation. The highest photocurrent density of 2.08 mA/cm^2^ at 1.23 V_RHE_ was obtained in the electrolyte without hole scavenger.^30^ Kubendhiran and co-workers proposed a BVO/zinc cobalt metal–organic framework (ZnCoMOF) composite as a photocatalyst for water oxidation. The BiVO_4_/ZnCoMOF photoanode shows a photocurrent density of 3.08 mA/cm^2^ at 1.23 V_RHE_, which is 4.21 times greater than that of the BVO photoanode.^31^ Wu et al. in situ-synthesized BVO coupled with the Co_3_(PO_4_)_2_ cocatalyst using a one-step solid-state process. A highest photocurrent density of 0.30 mA/cm^2^ at 1.23 V_RHE_ was obtained for BVO/Co_3_(PO_4_)_2_ while BVO only showed a photocurrent density of 0.13 mA/cm^2^ at 1.23 V_RHE_.^32^ Chen et al. synthesized five cheap cocatalysts based on nickel, cobalt, and iron to decorate on BVO photoanodes. The NiOOH/BiVO_4_ photoanode showed the highest photocurrent density of 2.10 mA/cm^2^ at 1.23 V_RHE_, while the BVO photoanode only shows a photocurrent density of 0.30 mA/cm^2^, owing to the better intrinsic properties of NiOOH and the continuous film on the BVO surface to improve connections between nanoparticles.^35^

Based on the high feasibility of applying the cocatalyst for promoting the photoelectrochemical catalytic ability, it is worth seeking for novel cocatalysts for the BVO system. It is worthy to note that limited reports investigating the photocharging abilities of metal oxides and applying these materials as the cocatalyst in the PEC water splitting systems. Nickel tellurium oxide (NTO) (Ni_3_TeO_6_, NTO) is a visible-light responsive semiconductor having a band gap of 2.48 eV.^36^ This bimetallic oxide shows a reduced band gap compared to its single metallic compounds, i.e., NiO with the band gap of 3.5 eV^37^ and TeO_2_ with the band gap of 3.64 eV.^38^ The nickel-based compound is earth-abundant, low cost, high catalytically active, and highly stabile in aqueous media during the PEC reaction.^39^ Singh et al. electrodeposited nickel oxide (NiO_*x*_) from [Ni(en)_3_]Cl_2_ (en = 1,2-diaminoethane, NiO_*x*_-en) in borate buffer (NaBi) solution as the water oxidation catalyst. The NiO_*x*_-en film sustained a current of 1.8 mA/cm^2^ for extended periods, compared with 1.2 mA/cm^2^ for films derived from [Ni(OH_2_)_6_](NO_3_)_2_ and [Ni(NH_3_)_6_]Cl_2_ in NaBi buffer.^40^ The tellurium has high electrical conductivity. Incorporating tellurium can improve charge transfer and further enhance the PEC catalytic ability. The high electrical conductivity of NTO also promoted its charge storage ability, which was verified in a previous work. Park et al. composited NTO with carbon nanotubes (NTO@CNTs) as a conversion-type anode for sodium-ion batteries. The NTO@CNTs exhibited a specific capacity of 495 mA h/g and high-rate performance up to 2000 mA/g.^41^ Gao et al. proposed a strategy to in situ form a NiB layer by tuning the composition of the neutral electrolyte with the additions of Ni and B species to improve the PEC performance of BVO photoanodes. The NiB/BVO photoanode exhibited a photocurrent density of 6.0 mA/cm^2^ at 1.23 V_RHE_ and an onset potential of 0.2 V_RHE_.^42^ Gao and co-workers utilized a plasma etching approach to reduce both interface/surface recombination at NiOOH/BVO and NiOOH/electrolyte junctions of the NiOOH/BVO junction as the photocatalyst for water oxidation.^43^ Several reports have studied the photoelectrocatalytic activity of NTO. Singh and Sharma proposed nickel doped α-bismuth oxide as a photocatalyst for the PEC study. It was verified that the Ni dopant can serve as shallow trapping energy sites for photoexcited charge carriers and improve the photoactivity.^44^ Iqbal et al. illustrated the photocharging and photoelectrocatalytic capabilities of NTO as the photoelectrocatalyst for the OER. The synthesized material has displayed an excellent charge storage capacity in KOH and Na_2_SO_4_ electrolytes.^45^ The cocatalytic ability of NTO has been proposed, but there are almost no reports investigating its cocatalytic function on the BVO system. It is worth understanding the feasibility of incorporating NTO as the cocatalyst for further enhancements on the photoelectrochemical catalytic ability of the BVO system.

In this work, NTO was first used as the cocatalyst for the BVO photoanode to accelerate the water splitting reactions in the PEC system. The NTO was synthesized by using the hydrothermal process. Different amounts of NTO were deposited on the BVO photoanode by using a simple drop casting method at room temperature. The material and photoelectrochemical properties of the NTO, BVO, and NTO/BVO systems were investigated. The highest photocurrent density of 6.05 mA/cm^2^@1.23 V_RHE_ was attained for the optimal NTO/BVO photoanode. The excellent long-term stability under continuous light illumination was obtained for NTO/BVO with photocurrent retention of 90.6% after 7000 s. The highly enhanced photoelectrochemical performance of NTO/BVO is primarily due to the higher light absorbance and production of electron carriers by incorporating the photocharging NTO. The water splitting mechanism of the NTO/BVO photoanode was also proposed based on the measured band positions and the possible interactions.

## 2 Experimental Section

### 2.1 Synthesis of the Ni_3_TeO_6_ Powder

**Scheme 1:**
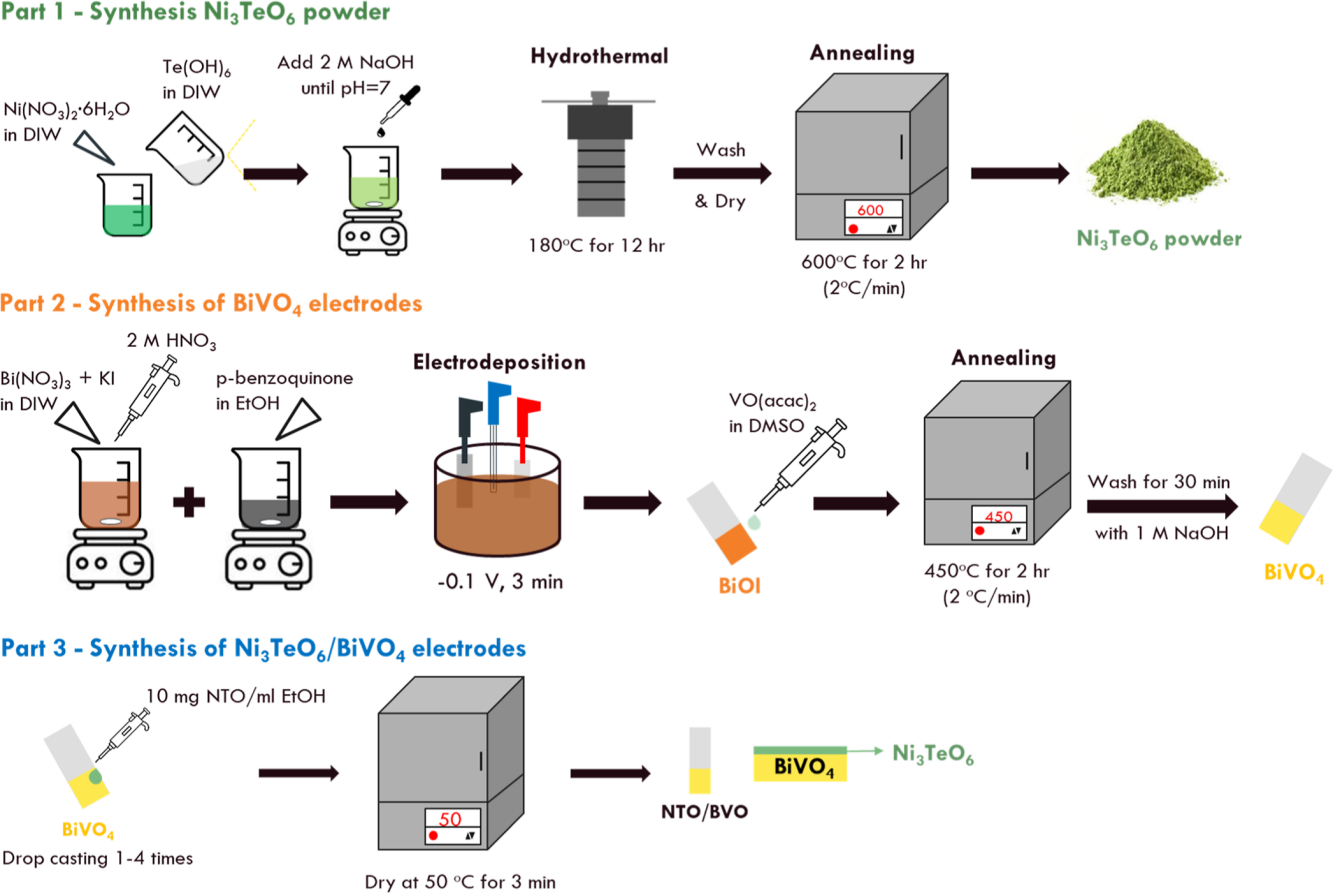
Experiments for Synthesizing NTO Powder, BVO Electrode, and NTO/BVO Electrodes

The Ni_3_TeO_6_ (NTO) powder was prepared by hydrothermal and annealing processes, as presented in Scheme 1. First, the precursor solution was prepared by dissolving 0.698 g of Ni(NO_3_)·6H_2_O (99.0%, Acros) and 0.184 g of Te(OH)_6_ (98%, Aladdin) in 30 mL of deionized water (DIW). Next, two solutions were mixed and stirred for 10 min, and the pH value of the solution was adjusted to 7 by adding 2 M NaOH. Second, the solution was transferred to a Teflon liner and put into the stainless-steel autoclave for conducting the hydrothermal process at 180 °C for 12 h. The mixture was washed for three times by DIW and ethanol and then centrifuged to collect the powder. Finally, the powder was annealed at 600 °C for 2 h at the heating rate of 2 °C/min.

### 2.2 Synthesis of Ni_3_TeO_6_/BiVO_4_ (NTO/BVO) Electrodes

Fabrication of the BiVO_4_ electrode is provided in the Supporting Information. The fabrication process for BVO and NTO/BVO electrodes are presented in Scheme 1. The Ni_3_TeO_6_/BiVO_4_ (NTO/BVO) electrodes were synthesized by a drop-casting method. The NTO solution was prepared by dispersing 0.05 g of NTO powder in 5 mL of ethanol. Subsequently, 10 μL of the NTO solution was drop cast on the BVO electrode for different times. The electrode was then dried at 50 °C for 3 min to obtain the NTO/BVO electrode. The NTO/BVO electrodes prepared by drop casting the NTO solution for 1, 2, 3, and 4 times were named as NTO/BVO-1, NTO/BVO-2, NTO/BVO-3, and NTO/BVO-4, respectively. The material and electrochemical characterization is described in the Supporting Information.

## 3 Results and Discussion

### 3.1 Material Analysis of BVO, NTO/BVO-1, NTO/BVO-2, NTO/BVO-3, and NTO/BVO-4

**Figure 1:**
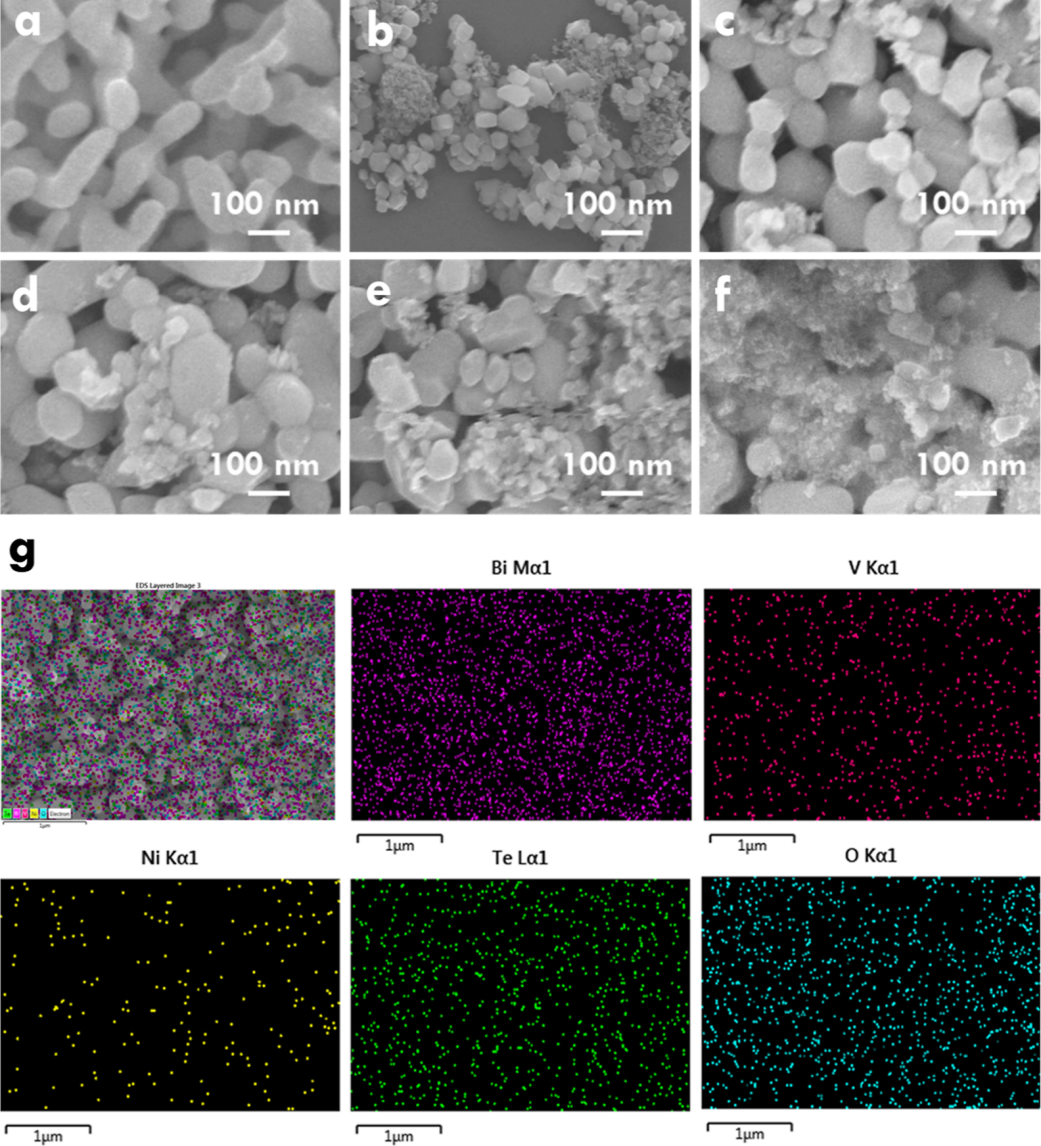
SEM images of (a) BVO, (b) NTO, (c) NTO/BVO-1, (d) NTO/BVO-2, (e) NTO/BVO-3, and (f) NTO/BVO-4; (g) EDX mapping spectra of NTO/BVO-2.

The morphology of BVO, NTO, NTO/BVO-1, NTO/BVO-2, NTO/BVO-3, and NTO/BVO-4 was first examined to understand the configurations of single materials and composites. Figure 1a presents the SEM image of the BVO photoanode, which shows the twisted rod-like structure arranged in an array. The top of the rods shows a width and length of around 70 and 150 nm, respectively. Figure 1b presents the SEM image of NTO. Two sizes of NTO were synthesized, including smaller particles with the size less than 10 nm and larger cubes having the size of around 30 to 50 nm. It was found that the size of NTO is much smaller than that of BVO, implying the high feasibility for decorating NTO on the surface of BVO. On the other hand, the SEM images of NTO/BVO-1, NTO/BVO-2, NTO/BVO-3, and NTO/BVO-4 are shown in Figure 1c–f, respectively. The obvious rodlike array and tiny particles were observed, which are possibly the BVO and NTO, respectively. It was also found that by using more times to deposit NTO on the BVO photoanode, higher amounts of the tiny particles appeared on the rodlike array. The coverage percentages of 11.6%, 33.5%, 53.1%, and 90.3% were, respectively, obtained for the NTO/BVO-1, NTO/BVO-2, NTO/BVO-3, and NTO/BVO-4, respectively. This phenomenon suggests the successful control of NTO deposition by simply varying the times for drop casting the NTO solution on the BVO photoanode. Figure 1g further presents the EDX mapping spectra of NTO/BVO-2. The elements of Bi, V, O, Ni, and Te were evenly distributed in the spectra. This result suggests the uniform deposition of NTO on the BVO photoanode.

**Figure 2:**
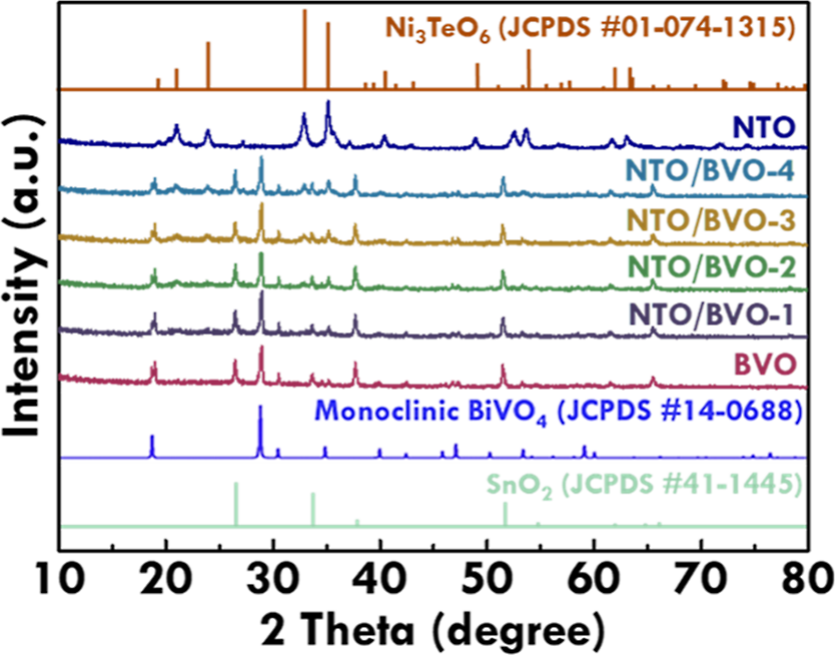
XRD patterns of BVO, NTO/BVO-1, NTO/BVO-2, NTO/BVO-3, NTO/BVO-4, and NTO.

The compositions of BVO, NTO, NTO/BVO-1, NTO/BVO-2, NTO/BVO-3, and NTO/BVO-4 were then analyzed by using the XRD patterns, as presented in Figure 2. The standard patterns of SnO_2_ (JCPDS #41-1445),^46^ monoclinic BiVO_4_ (JCPDS #14-0688),^33^ and Ni_3_TeO_6_ (JCPDS #01-074-1315)^33^ were also placed in this figure for examining the compositions. The patterns of BVO, NTO/BVO-1, NTO/BVO-2, NTO/BVO-3, and NTO/BVO-4 show the signals corresponding to SnO_2_, which were attributed from the FTO glass. The NTO was examined using the powder form, so the peaks corresponding to SnO_2_ are not observed in the pattern of NTO. The peaks corresponding to the monoclinic BiVO_4_ were obtained in the patterns of BVO, NTO/BVO-1, NTO/BVO-2, NTO/BVO-3, and NTO/BVO-4, suggesting the successful synthesis of monoclinic BiVO_4_ and the limited changes on phase and composition of BiVO_4_ after depositing NTO on the surface by drop casting several times. The pattern of the NTO powder shows perfect matches to the standard pattern of Ni_3_TeO_6_, indicating the successful synthesis of Ni_3_TeO_6_ using a hydrothermal process. On the other hand, the peaks corresponding to NTO at the 2θ of 20.4°, 20.9°, 23.8°, 32.8°, 35.2°, 40.5°, 52.5°, and 53.6° were observed in the patterns of NTO/BVO-1, NTO/BVO-2, NTO/BVO-3, and NTO/BVO-4, implying the successful incorporation of NTO in the photoanodes. There are almost no BVO peak shifts, which can be observed in the patterns of the pure BVO and the NTO/BVO composites. Therefore, the combination of BVO and NTO is considered as the simple physical deposition with limited interactions in bonding.

**Figure 3:**
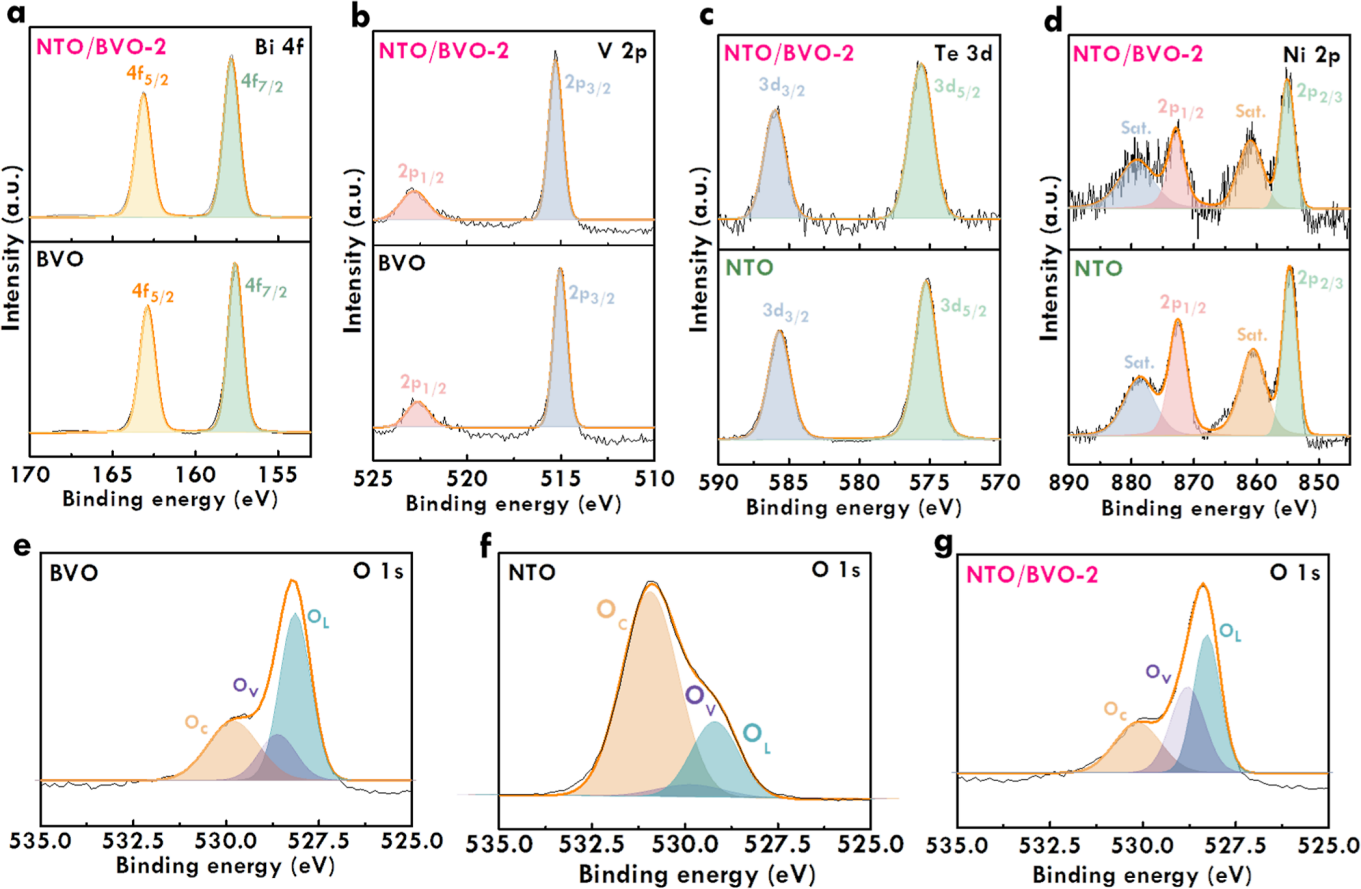
(a) Bi 4f, (b) V 2p, (c) Te 3d, and (d) Ni 2p spectra of BVO and NTO/BVO-2; O 1s spectra of (e) BVO, (f) NTO, and (g) NTO/BVO-2.

To further investigate the electronic configuration and binding energy in the systems of BVO, NTO, and NTO/BVO-2, XPS spectra were measured. Figure 3a shows the Bi 4f spectra for BVO and NTO/BVO-2. Two peaks were observed in the Bi 4f spectra for BVO and NTO/BVO-2 at similar binding energy. These peaks were fitted as 4f_3/2_ and 4f_5/2_ orbitals, respectively, located at 163.1 and 157.8 eV. The V 2p spectra for BVO and NTO/BVO-2 are shown in Figure 3b, which presents two peaks at similar binding energy in the two spectra. The two peaks were fitted as 2p_1/2_ and 2p_3/2_ orbitals located at 522.9 and 515.3 eV, respectively. The similar binding energies of the Bi 4f and V 2p orbitals for BVO and NTO/BVO-2 indicate that the intrinsic properties of bismuth and vanadium are not influenced by the deposition of NTO using the simple drop casting technique. The Te 3d spectra of NTO and NTO/BVO-2 are shown in Figure 3c, which shows two distinguish peaks characterized as 3d_3/2_ and 3d_5/2_ orbitals, respectively, at the binding energy of 586.1 and 575.6 eV. The Ni 2p spectra of NTO and NTO/BVO-2 are shown in Figure 3d, which was characterized as 2p_1/2_ and 2p_3/2_ peaks followed by satellite peaks. The 2p_1/2_ and 2p_3/2_ peaks were fitted at binding energies of 873.0 and 855.1 eV, respectively. The similar binding energy of Te 3d and Ni 2p orbitals for NTO and NTO/BVO-2 imply the limited influences on NTO with and without depositing on the BVO surface. The similar configurations of the Bi 4f, V 2p, Te 3d, and Ni 2p spectra for BVO, NTO, and NTO/BVO-2 imply the simple physical combination of BVO and NTO in the composite. In turn, the O 1s spectra of BVO, NTO, and NTO/BVO-2 are shown in Figure 3e–g, respectively. Three peaks corresponding to lattice oxygen (O_L_), oxygen vacancy (O_V_), and chemical adsorbed oxygen (O_C_) were characterized in the O 1s spectra. The peak configurations in the O 1s spectra of BVO and NTO/BVO-2 are quite similar, suggesting that oxygen is mainly contributed from BVO in the NTO/BVO-2 system. To compare the O 1s spectra of BVO and NTO/BVO-2, it was found that the peak area of O_V_ is much larger in the spectrum of NTO/BVO-2. Generally, the O_V_ commonly plays as the active sites for catalyzing OER. The large enhancements of O_V_ in the NTO/BVO composite is favorable for designing an efficient photoelectrochemical catalyst toward OER.

**Figure 4:**
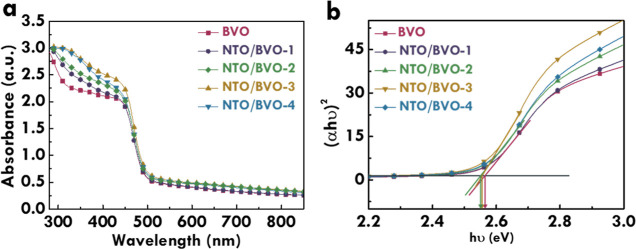
(a) UV–vis spectra and (b) Tauc plot of BVO, NTO/BVO-1, NTO/BVO-2, NTO/BVO-3, and NTO/BVO-4.

The optical properties of BVO, NTO/BVO-1, NTO/BVO-2, NTO/BVO-3, and NTO/BVO-4 were then examined by the UV–vis spectra, as shown in Figure 4a. The light absorbance spectra for all samples present the edges at about 500 nm, which is the characteristic light absorbance for monoclinic BiVO_4_. The similar light absorbance edges for BVO and the NTO/BVO composites suggest that the optical features and band structures of BVO are limitedly influenced after depositing NTO with the drop casting technique. On the other hand, the light absorbance of all NTO/BVO photoanodes is higher than that of BVO, suggesting that the incorporation of NTO can improve the light absorbance and enhance the utilization of incident light. The excitation of light-induced electrons in NTO is from O 2p to empty Ni 3d orbitals, resulting in the optical absorbance at near 500 nm.^36^ To compare the light absorbance of the NTO/BVO photoanodes, it was found that the light absorbance of the resulting NTO/BVO photoanode was increased with more deposition of NTO. Based on the similar light-absorbed abilities of BVO and NTO, this phenomenon is attributed to the larger exposure of the photocatalyst to incident light by the smaller particle sizes of NTO. As a result, the largest light absorbance was achieved for the NTO/BVO-3 photoanode. However, by depositing the NTO in the BVO photoanode four times, the NTO/BVO-4 photoanode presented a reduction in the light absorbance compared to that for the NTO/BVO-3 photoanode. This phenomenon is due to the over coverage of NTO on BVO. Since the BVO presents the array configuration, the over coverage of NTO may hinder the light absorbance at the side wall of the rods. Also, the incident light may be unable to transmit through the gaps between rods when too many NTO particles were deposited on the surface of BVO. Moreover, the band configurations of BVO, NTO/BVO-1, NTO/BVO-2, NTO/BVO-3, and NTO/BVO-4 photoanodes were examined by the Tauc plots, as presented in Figure 4b. The Tauc plot was obtained from the light absorbance spectra and the Tauc equation.^23^ The band gap of materials was estimated via the two tangent lines shown in this figure. The *x* value of the intersected point is the band gap of the semiconductor. The similar band gaps of 2.5 eV were obtained for BVO and NTO/BVO systems. As reported by previous studies, the band gaps of BVO and NTO are around 2.4^47^ and 2.88 eV,^48^ respectively. Therefore, the band gaps of the NTO/BVO system are more like the band gap of BVO. This result is possibly due to the much larger occupation of BVO in the composite and the higher contributions on the light absorbance from BVO than from NTO.

### 3.2 Photoelectrochemical Analysis of BVO, NTO/BVO-1, NTO/BVO-2, NTO/BVO-3, NTO/BVO-4, and NTO Photoanodes

**Figure 5:**
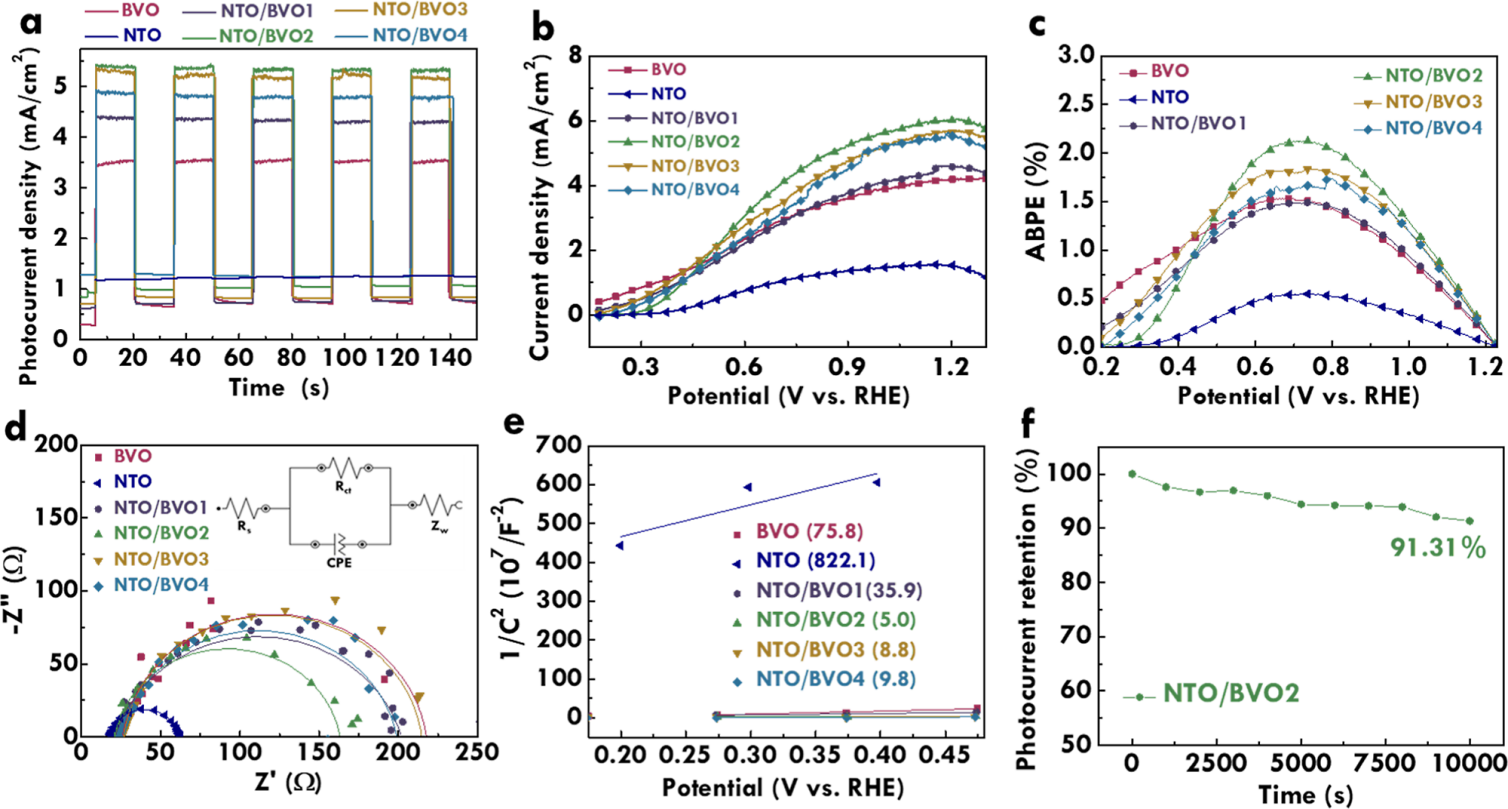
(a) Transient current plot at 1.23 V_RHE_, (b) LSV curves at 20 mV/s under light illumination, (c) ABPE plot, (d) Nyquist plot at 1.23 V_RHE_ under light illumination, (e) Mott–Schottky plot for BVO and NTO/BVO-2 photoanodes, and (f) photocurrent retention as a function of time (stability test) for the NTO/BVO-2 photoanode.

To investigate the photoelectrocatalytic ability of BVO, NTO/BVO-1, NTO/BVO-2, NTO/BVO-3, NTO/BVO-4, and NTO electrodes, the photoelectrochemical performance was then analyzed. The photoresponsive behavior was first examined. Figure 5a presents the chopped chronoamperometry of BVO, NTO/BVO, and NTO electrodes. The potential was set at 1.23 V_RHE_. This figure was obtained by turning on and off the light repeatedly during the measurement of the current density. It was found that the current density enhanced to the largest value in very short moments when the light was turned on. Similarly, when light was turned off, the current density suddenly decreased to the smallest value. These phenomena verify the photoresponsive behavior of BVO and all NTO/BVO photoanodes. On the other hand, almost no spikes are observed in the curves for BVO and all NTO/BVO photoanodes. The spikes originated from the electron and hole recombination. The limited spikes in the curves of BVO and all NTO/BVO photoanodes are favorable for an excellent photocatalyst toward water oxidation. The limited charge recombination was achieved in the BVO photoanode, and even after depositing by NTO the charge recombination was not enhanced. The great contacts between NTO and BVO were therefore proved. It is quite advantageous to use a very simple drop casting technique for depositing NTO on BVO without generating too many defects for inducing serious charge recombination. In addition, the NTO electrode shows almost no response to the incident light, indicating that the main light absorber is BVO not NTO. The current of the NTO electrode remained almost 1 mA/cm^2^, which is like the current density of the NTO/BVO-4 electrode in the dark condition. This phenomenon again suggests the electrocatalyst ability of NTO which was also displayed in the NTO/BVO composite.

Moreover, the linear sweep voltammetry (LSV) curves of BVO, NTO/BVO-1, NTO/BVO-2, NTO/BVO-3, NTO/BVO-4, and NTO electrodes were measured to understand their photoelectrocatalytic performances toward the OER, as shown in Figure 5b. The photocurrent density at 1.23 V_RHE_ and the onset potential of BVO, NTO/BVO, and NTO electrodes are listed in Table 1. The photocurrent density of 4.19, 4.58, 6.05, 5.66, 5.45, and 1.44 mA/cm^2^ was achieved for the BVO, NTO/BVO-1, NTO/BVO-2, NTO/BVO-3, NTO/BVO-4, and NTO electrodes, respectively. The BVO presented a smaller photocurrent density than those for all of the NTO/BVO photoanodes. This result suggests the advantages of combining NTO on the surface of BVO to improve the photoelectrocatalytic abilities. The NTO may play as the cocatalyst for providing the hole sink and reducing activation energy. More OER could occur at the cocatalyst, and more efficient water splitting may be induced in the cocatalyst/photocatalyst system. Among the NTO/BVO photoanodes, the photocurrent density is larger for the NTO/BVO photoanode prepared with more depositions of NTO. The largest photocurrent density of 6.05 mA/cm^2^ was achieved for the NTO/BVO-2 photoanode, owing to the suitable amounts of NTO deposition with the high optical responses and abundant active sites. However, further increasing the deposition of NTO leads to the reduction of the photocurrent density for the resulting NTO/BVO photoanodes. That is, the photocurrent densities of NTO/BVO-3 and NTO/BVO-4 photoanodes are smaller than those of the NTO/BVO-2 photoanode. The balances between optical and electrical features play significant roles in the photoelectrochemical performance. The deposition of NTO on BVO can not only provide hole sinks for conducting the OER but also generate grain boundaries in-between to cause charge recombination. Therefore, the effect of the generated grain boundaries may be more dominant comparing the enhanced number of hole sinks by depositing too many NTO particles on the BVO surface. As a result, worse photocatalytic abilities were obtained for NTO/BVO-3 and NTO/BVO-4 compared to NTO/BVO-2. On the other hand, the onset potentials of 0.24, 0.26, 0.25, 0.27, and 0.27 V_RHE_ were achieved for the BVO, NTO/BVO-1, NTO/BVO-2, NTO/BVO-3, and NTO/BVO-4 photoanodes, respectively. The similar onset potentials for BVO and NTO/BVO suggest that the charge recombination and driving force required for initiating the OER are similar to those without NTO deposition. Furthermore, the applied bias photon-to-current efficiency (ABPE) plots as the function of potential for BVO, NTO/BVO-1, NTO/BVO-2, NTO/BVO-3, NTO/BVO-4, and NTO electrodes are shown in Figure 5c. The ABPE plots were obtained from the LSV curves. The ABPE values of 1.54%@0.67 V_RHE_, 1.50%@0.74 V_RHE_, 2.13%@0.72 V_RHE_, 1.84%@0.74 V_RHE_, 1.75%@0.79 V_RHE_, and 0.55%@0.72 V_RHE_ were achieved for the BVO, NTO/BVO-1, NTO/BVO-2, NTO/BVO-3, NTO/BVO-4, and NTO electrodes, respectively. The largest ABPE value of 2.13% was obtained for the NTO/BVO-2 photoanode, implying that the suitable incorporation of NTO with the high photoresponsive and electron sink properties in the BVO photoanode can more effectively increase the conversion of incident light to charges.

**Table 1:** Photocurrent Density, Onset Potential, Maximum ABPE, and *R*_CT_ Values of BVO, NTO/BVO-1, NTO/BVO-2, NTO/BVO-3, and NTO/BVO-4 Photoanodes^a^

electrode	photocurrent density (mA/cm^2^@1.23 V_RHE_)	onset potential (V_RHE_)	*R*_CT_ (Ω)
BVO	4.19	0.24	191.56
NTO/BVO-1	4.58	0.25	179.90
NTO/BVO-2	6.05	0.33	141.94
NTO/BVO-3	5.66	0.27	187.80
NTO/BVO-4	5.45	0.27	173.82

a Three samples were measured for each parameter to examine the reliability of the experiments.

Furthermore, the system resistances in the BVO, NTO/BVO-1, NTO/BVO-2, NTO/BVO-3, NTO/BVO-4, and NTO electrodes were analyzed using Nyquist plots, as shown in Figure 5d. The equivalent circuit was also shown in this plot to fit the charge-transfer resistances (*R*_CT_), which is the resistance to hinder charge transfer at the electrode and electrolyte interface.^49^ The *R*_CT_ value of BVO, NTO/BVO, and NTO electrodes is shown in Table 1. The R_CT_ values of 191.56, 179.90, 141.94, 187.80, 173.82, and 47.21 Ω were, respectively, obtained for the BVO, NTO/BVO-1, NTO/BVO-2, NTO/BVO-3, NTO/BVO-4, and NTO electrodes. The *R*_CT_ value of the BVO photoanode is larger than those of the NTO/BVO photoanodes, implying that by depositing the cocatalyst of NTO on BVO the band aliments can be established to accelerate charge transfer and reduce transporting obstacles. Among the NTO/BVO photoanodes, the lowest *R*_CT_ value was obtained for the NTO/BVO-2 photoanode. It was indicated that a suitable amount of NTO depositing on the surface of BVO can develop efficient charge cascades and prohibit sacrifices of light absorbance by BVO. Hence, the largest photocurrent density and the lowest charge-transfer resistance for NTO/BVO-2 are beneficial for designing an excellent photoelectrical catalyst toward the OER.

In addition, the carrier density of BVO, NTO/BVO-1, NTO/BVO-2, NTO/BVO-3, NTO/BVO-4, and NTO electrodes was evaluated via Mott–Schottky plots, as presented in Figure 5e. The plots were fitted as presented in this figure. As is known, the slope of the fitted line is inversely proportional to the carrier density in the electrode. The slope values of 75.8, 39.9, 5.0, 8.8, 9.8, and 822.1 were, respectively, obtained for the BVO, NTO/BVO-1, NTO/BVO-2, NTO/BVO-3, NTO/BVO-4, and NTO electrodes. Hence, the carrier densities of 2.74 × 10^23^, 5.20 × 10^23^, 4.12 × 10^24^, 2.37 × 10^24^, 2.13 × 10^24^, and 5.16 × 10^22^ cm^–3^ were obtained for the BVO, NTO/BVO-1, NTO/BVO-2, NTO/BVO-3, NTO/BVO-4, and NTO electrodes, respectively. The carrier density of the BVO photoanode is smaller than that of the NTO/BVO photoanodes. The incorporation of NTO into the BVO photoanode is verified to enhance the generation of charge carriers. Among the NTO/BVO photoanodes, the NTO/BVO-2 photoanode shows the highest carrier density, indicating that the suitable deposition amount of NTO can also induce the largest amounts of charges.

Last but not least, the photoelectrochemical catalytic stability of the NTO/BVO-2 photoanode was examined by using the chronoamperometry method. The applied potential is 1.23 V_RHE_. The light illumination was conducted for 10,000 s. Figure 5f presents the relation of the photocurrent density of the NTO/BVO-2 photoanode to the illuminating duration. The photocurrent retention of 91.31% was achieved after continuously illuminating the photoanode for 10,000 s. The very high photocurrent retention for the NTO/BVO-2 photoanode verifies the great photoelectrochemical catalytic stability, which is one of the significant factors in evaluating the performance of a photoanode. The morphology changes for BVO and NTO/BVO photoanodes after the stability test for 10,000 s were also evaluated. Figure S1a–e in the Supporting Information shows the SEM images of BVO, NTO/BVO-1, NTO/BVO-2, NTO/BVO-3, and NTO/BVO-4 photoanodes after the stability test. These images are highly like those without stability test (Figure 1), implying the high maintenance of morphology and NTO distribution after the stability test for all samples.

The charge separation efficiency in BVO and NTO/BVO-2 electrodes was quantitatively assessed by using the photocurrent from water and sulfite oxidation. The reactions for water oxidation and sulfite oxidation are, respectively, shown in eqs 1 and 2 as follows.
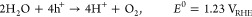
1
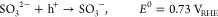
2

**Figure 6:**
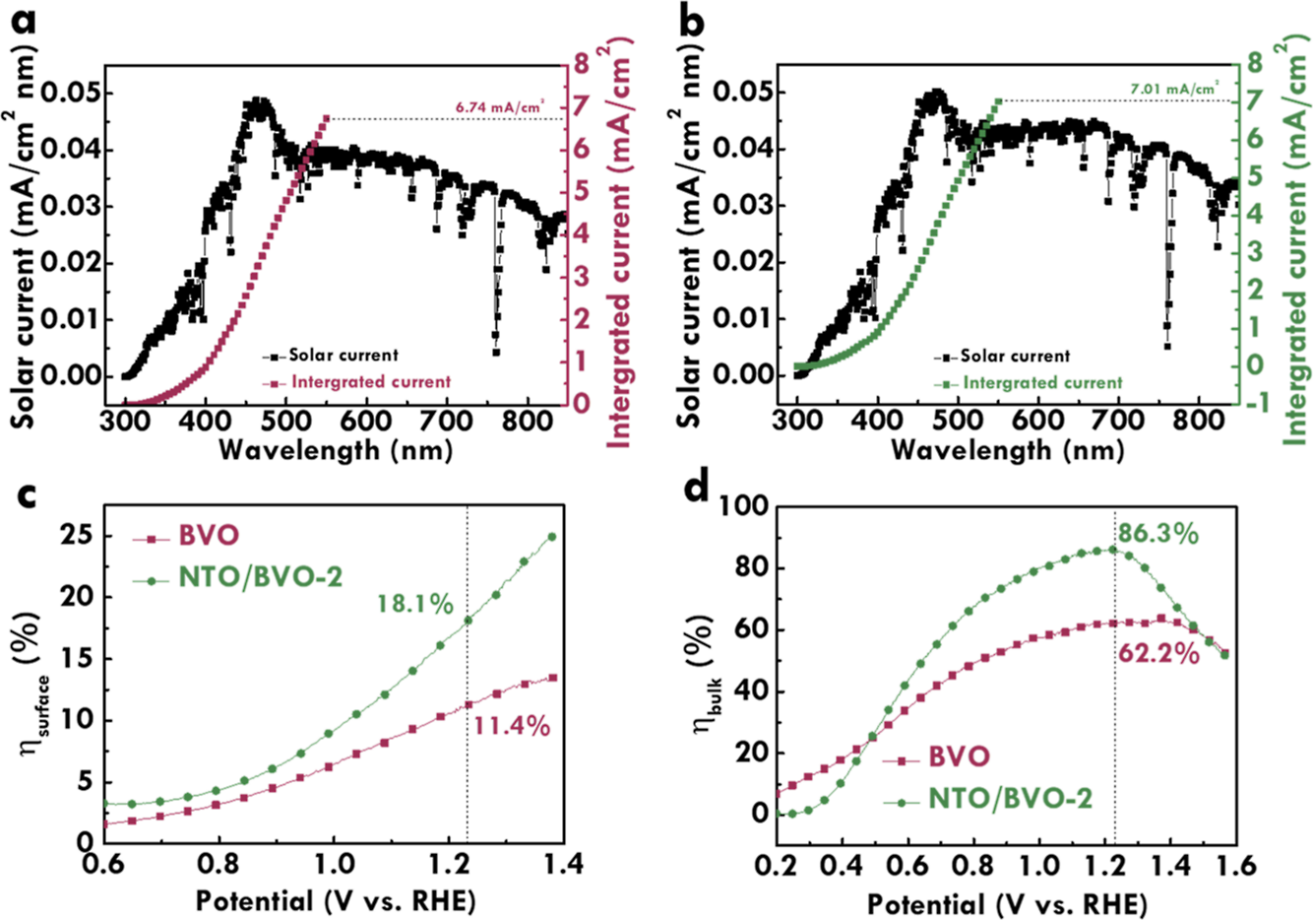
LHE corresponding to AM 1.5G spectrum of (a) BVO and (b) NTO/BVO-2 electrodes; (c) surface charge separation efficiency and (d) bulk charge separation efficiency as a function of potentials for BVO and NTO/BVO-2 anodes.

The charge-transfer photocurrent densities (*J*_abs_) of the electrodes were obtained by integrating the optical measurements with the standard solar spectrum using eq 3 as follows.
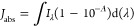
3with *I*_λ_ is the light intensity at a specific wavelength λ and *A* is the absorption coefficient. Figure 6a,b shows the light harvesting efficiency (LHE) corresponding to the AM 1.5G spectrum of BVO and NTO/BVO-2 electrodes, respectively. Light absorption by a photocatalyst generates *J*_abs_ that encounters two main losses of bulk and surface recombination, so the measured photocurrent during water oxidation (*J*_H_2_O_) is expressed by *J*_H_2_O_ = *J*_abs_ × η_bulk_ × η_surf_, where η_bulk_ and η_surf_ are, respectively, the bulk and surface charge separation efficiencies. Since the surface charge separation yield of SO_3_^2–^ is approaching 100%, that is, η_surf_ equals to 1, the photocurrent from sulfite oxidation (*J*_SO_3__) is expressed as *J*_SO_3__ = *J*_abs_ × η_bulk_. Therefore, the η_bulk_ and η_surf_ were, respectively, calculated by using eqs 4 and 5 as follows.
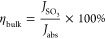
4
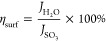
5

The surface and bulk separation efficiencies of BVO and NTO/BVO-2 photoanodes as a function of potentials are, respectively, shown in Figure 6c,d. The surface separation efficiencies of 11.4 and 18.1% were, respectively, obtained for the BVO and NTO/BVO-2 photoanodes. Also, the bulk separation efficiencies of 62.2 and 86.3% were, respectively, achieved for the BVO and NTO/BVO-2 photoanodes. The higher surface and bulk separation efficiencies of NTO/BVO-2 compared to those of BVO indicate the feasibility of incorporating NTO to enhance the photocatalytic ability toward water oxidation.

**Figure 7:**
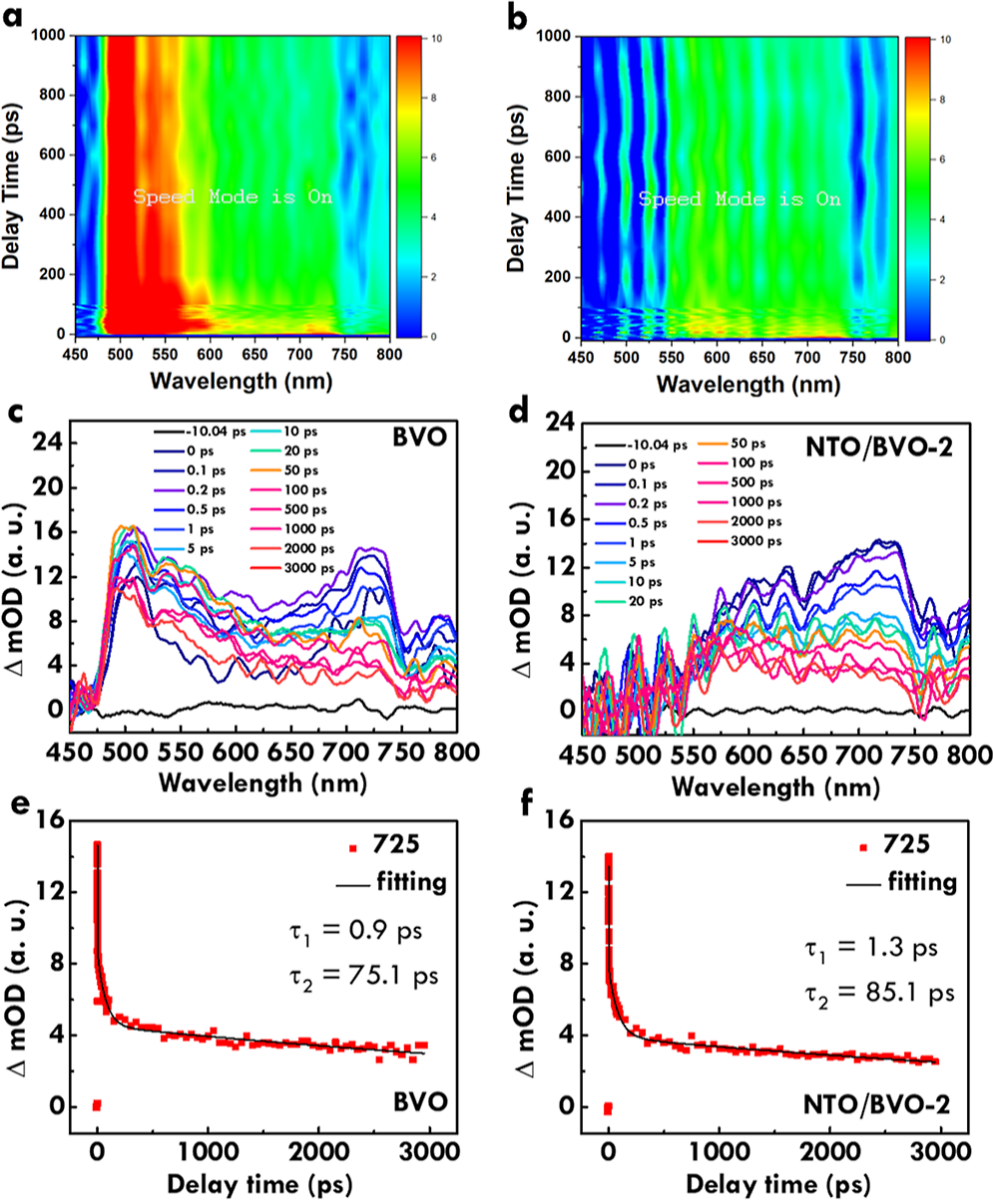
Three-dimensional contour plots of TAS observation for (a) BVO and (b) NTO/BVO-2; TAS spectra of (c) BVO and (d) NTO/BVO-2; and the corresponding kinetics decay at 725 nm for (e) BVO and (f) NTO/BVO-2.

The charge carrier dynamics was further evaluated by transient absorption spectroscopy (TAS). Figure 7a,b shows the three-dimensional contour plots of TAS observation for BVO and NTO/BVO-2. The TAS spectra of BVO and NTO/BVO-2 are, respectively, shown in Figure 7c,d. In addition, the kinetic decay curves of BVO and NTO/BVO-2 were recorded and fitted by the three-exponential function. The corresponding kinetics decays at 725 nm for BVO and NTO/BVO-2 are, respectively, presented in Figure 7e,f. The τ_1_ and τ_2_ represent the observed slow decay component and the fast recombination of excited holes and electrons, respectively. The BVO and NTO/BVO-2, respectively, show τ_1_ values of 0.9 and 1.3 ps, while τ_2_ values of 75.1 and 85.1 ps were obtained for BVO and NTO/BVO-2. The NTO/BVO-2 shows faster trapping electrons and slower recombination of photogenerated carriers compared to BVO, implying the more photoexcited electrons captured by trap states in NTO/BVO-2. This result contributes to the higher carrier separation and transfer efficiency of NTO/BVO-2.

To fairly compare the photoelectrochemical catalytic ability toward water oxidation, the photocatalysts, electrolyte, pH value of electrolyte, and photocurrent density of BVO based photoanode reported in previous literature and the present work are listed in Table 2. Several bimetallic oxides were applied as the cocatalyst in the BVO photoanodes, such as nickel/cobalt, nickel/iron, and cobalt/iron. The single metallic compounds, such as nickel oxide and cobalt phosphide, were also incorporated into the BVO photoanodes for catalyzing water oxidation. The photocurrent density of 6.05 mA/cm^2^ achieved in the present work is much higher than those of the similar systems reported in previous studies. The comparisons with previous studies reveal that the Ni_3_TeO_6_/BiVO_4_ newly proposed in this work shows outstanding photoelectrochemical performance toward photoelectrochemical catalyzing water oxidation.

**Table 2:** Partial Lists of Photocatalysts, Electrolyte, pH Value of the Electrolyte, and Photocurrent Density of BVO-Based Photoanode Reported in Previous Literature and the Present Work

photoanode	electrolyte	pH value	photocurrent density at 1.23 V_RHE_ (mA/cm^2^)	refs
Ni _0.4_Co_0.6_O/BiVO_4_	0.5 M Na_2_SO_4_		3.20	(50)
NiFeO_*x*_/BiVO_4_	0.5 M borate buffer solution	9.5	4.17	(51)
CoFe_2_O_4_-BiVO_4_	0.2 M Na_2_SO_4_	7.0	4.43	(52)
NiO/BiVO_4_	0.5 M Na_2_SO_4_		1.94	(53)
N-CoFeO/BiVO_4_	1 M BK_3_O_3_	9.7	4.83	(54)
BiVO_4_/Bi_2_S_3_/NiCoO_2_	0.5 M Na_2_SO_4_	6.5	2.58	(55)
BiVO_4_/NiCoO_2_	0.1 M potassium phosphate buffered	7.0	3.60	(56)
CoP/BiVO_4_:WO_3_	0.5 M Na_2_SO_4_	6.5	2.81	(57)
Ni_3_TeO_6_/BiVO_4_	0.5 M Na_2_SO_4_ and 0.5 M Na_2_SO_3_	9.8	6.05	this work

### 3.3 Band Configuration Analysis and the OER Catalytic Mechanisms of NTO/BVO

**Figure 8:**
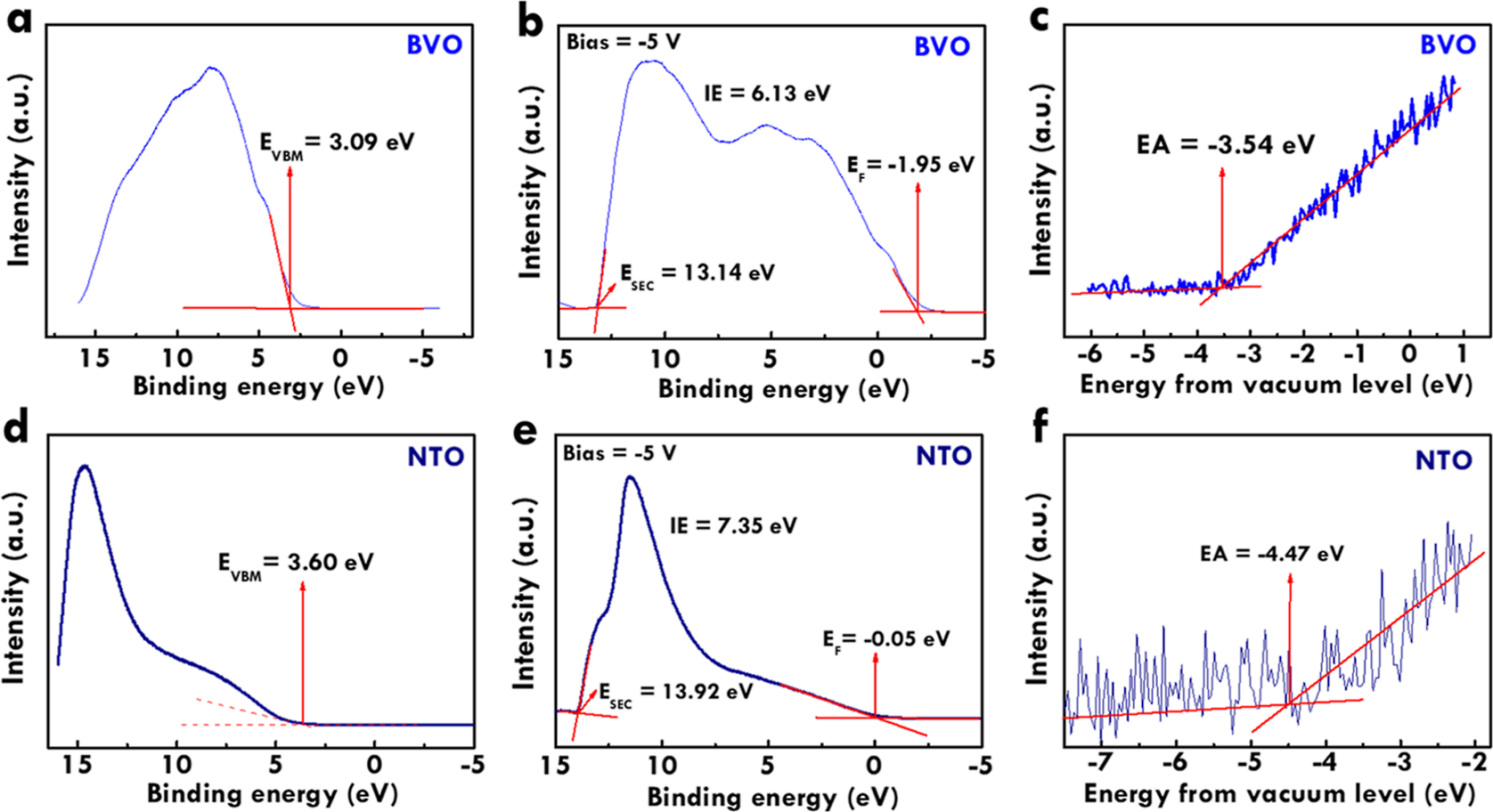
(a) Valence band region without bias, (b) valence band region measured with the bias of −5 V, and (c) secondary cutoff region for BVO; (d) valence band region without bias, (e) valence band region measured with the bias of −5 V, and (f) secondary cutoff region for NTO. The energy of 21.22 eV (*h*ν) was applied for the measurements.

To understand the band edges of BVO and NTO and discuss the possible mechanism, Figure 8a–c, respectively, shows the valence band region without bias, valence band region measured with the bias of −5 V and secondary cutoff region for BVO. At the same time, the valence band region without bias, valence band region measured with the bias of −5 V, and secondary cutoff region for NTO are presented in Figure 8d–f, respectively. The conduction band minimum and valence band maximum of BVO and NTO were calculated as follows. It should be noted that *E*_VBM_ is the energy level of valence band maximum, *E*_SEC_ is the effective secondary electron emission coefficient, *E*_F_ is the Fermi level energy, *E*_VAC_ is the energy level of vacuum, and *E*_CBM_ is the energy level of conduction band minimum. For BVO, an *E*_VBM_ of 3.09 eV is obtained from Figure 8a. The ionization energy (IE) was calculated by *h*ν (*E*_SEC_ – *E*_F_), which equals 6.13 eV. The values of *E*_SEC_ and *E*_F_ are obtained from Figure 8b. The *E*_VAC_ is equal to *E*_VBM_ – IE, which is −3.04 eV. In turn, the electron affinity (EA) is obtained from Figure 8c. The value of EA is found to be −3.54 eV. Last, the *E*_CBM_ is equal to *E*_VAC_ – EA, which is 0.50 eV. To check, the band gap is equal to *E*_VBM_ – *E*_CBM_ which is 2.59 eV. This band gap is similar to that obtained from the light absorbance spectrum. Similar calculations were applied for NTO, and the *E*_VBM_ of 3.60 eV is obtained from Figure 8d. The IE was calculated by *h*ν – (*E*_SEC_ – *E*_F_), which equals to 7.35 eV. The values of *E*_SEC_ and *E*_F_ are from Figure 8e. The *E*_VAC_ is equal to *E*_VBM_ – IE, which is −3.75 eV. In turn, the EA is obtained from Figure 8f. The value of EA is found to be −4.47 eV. Last, the *E*_CBM_ is equal to *E*_VAC_ – EA, which is 0.72 eV. To check, the band gap is equal to *E*_VBM_ – *E*_CBM_ which is 2.88 eV. This band gap is similar to that obtained from the light absorbance spectrum.

**Scheme 2:**
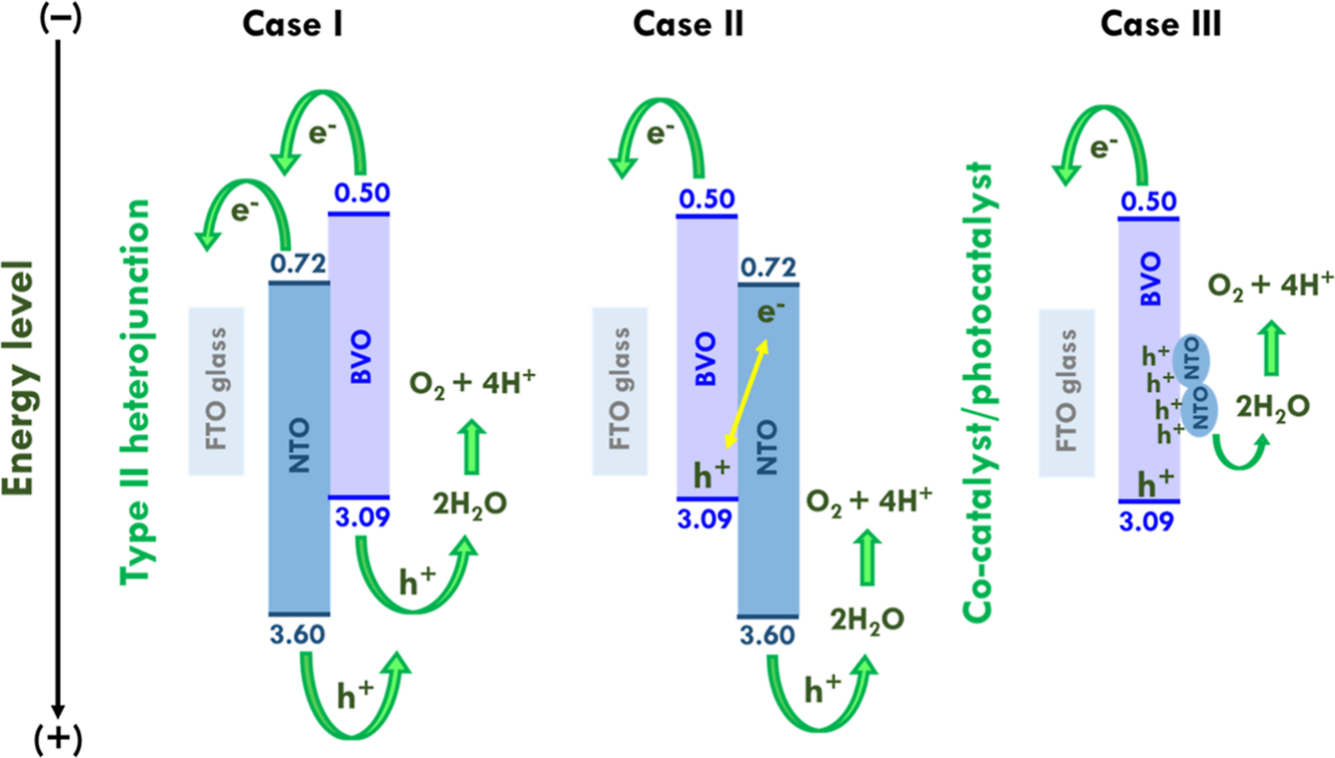
Illustration of Band Positions of NTO and BVO Combined in Different Sequences and Functions

Scheme 2 shows an illustration of band positions of NTO and BVO combined in different sequences and functions. Three cases were presented to discuss the possible combinations of NTO and BVO as well as the functions of them. Case I shows the inverse sequence of the photoanode fabricated in this work. The band positions of NTO and BVO establish the type II heterojunction in this sequence. That is, depositing NTO on the FTO glass prior to the growth of BVO. Case II shows the same sequence of the photoanode fabricated in this work. The band positions of NTO and BVO cannot establish the perfect type II heterojunction to transfer electrons from NTO and BVO to the FTO glass, and to transfer holes from BVO and NTO to the electrolyte. Therefore, the large enhancements on the photoelectrochemical catalytic ability of NTO/BVO-2 proposed in this study is inferred to result from the cocatalyst/photocatalyst system, as presented in Case III. The NTO acts as the cocatalyst to attract holes. The hole sink can accelerate the water oxidation and realize better photoelectrochemical catalytic ability. Therefore, the possible mechanism for this NTO/BVO photoanode is the cocatalyst/photocatalyst system. The light illuminated on the BVO photocatalyst, and abundant electrons and holes were generated at the same time. The electrons went through the CBM of BVO to that of the FTO glass. The mot portions of holes went to NTO to generate a hole sink. The water in the electrolyte would primarily react with the holes in NTO since the high concentration of holes were gathered in this hole sink. As a result, the greater water oxidation reaction could happen at these cocatalysts. The NTO can also be illuminated by the incident light and generate charges. The holes produced by NTO can directly catalyze OER. Last, the electrons and holes generated by NTO and BVO can possibly combine to reduce the charge recombination with the electrons and holes generated by BVO and NTO, respectively.

## 4 Conclusions

NTO was first proposed as the cocatalyst for depositing on the BVO photoanode as the cocatalyst/photocatalyst system for water oxidation. Different amounts of NTO were deposited on the BVO photoanode by using a drop casting technique. The interaction between NTO and BVO was verified to be a simple physical combination. The larger light absorbance was achieved for the NTO/BVO photoanode compared to that for the pure BVO photoanode. The largest photocurrent density of 6.05 mA/cm^2^ was obtained at 1.23 V_RHE_ for the NTO/BVO-2 photoanode, owing to the suitable amount of NTO deposition to induce high light absorbance, abundant oxygen vacancies, and great carrier density. This optimal NTO/BVO photoanode also shows the largest ABPE value and the smallest charge-transfer resistance. This NTO/BVO-2 photoanode also presents excellent photoelectrochemical catalytic stability. The photocurrent retention of 91.31% was attained after continuously illuminating the system for 10,000 s. More surface engineering on NTO/BVO with parameter optimizations will be realized to seek more excellent photoelectrochemical catalytic ability. The optimization of thickness for the NTO and BVO layers can be further optimized to improve the photoelectrochemical catalytic ability toward water oxidation in the near future.

## Supporting Information Available

Measurements and characterizations, and SEM images of BVO, NTO/BVO-1, NTO/BVO-2, NTO/BVO-3, and NTO/BVO-4 after the stability test (PDF)
