# Supplementary material for: Facile Combination of Bismuth Vanadate with Nickel Tellurium Oxide for Efficient Photoelectrochemical Catalysis of Water Oxidation Reactions
Source: ACS Appl Mater Interfaces. 2024 Sep 5;16(37):49249–61. doi: 10.1021/acsami.4c07117 (PMC11420875; doi:10.1021/acsami.4c07117)
Supplement: Supplementary file 1 — am4c07117_si_001.pdf [file am4c07117_si_001.pdf]

Supporting information

Facile Combination of Bismuth Vanadate with Nickel Tellurium Oxide for Efficient Photoelectrochemical Catalysis of Water Oxidation Reactions

Yu-Hsuan Chiu[a], Ren-Jei Chung[a], Chutima Kongvarhodom[b,c], Muhammad Saukani[d], Sibidou Yougbaré[e], Hung-Ming Chen[f], Yung-Fu Wu[g*] and Lu-Yin Lin[a*]

[a]Department of Chemical Engineering and Biotechnology, National Taipei University of Technology, Taipei, Taiwan

[b]Department of Chemical Engineering, King Mongkut's University of Technology Thonburi, 126 Pracha-u-thit, Toong-kru, Bangkok 10140, Thailand

[c]Department of Chemical Engineering, University of New Brunswick, Fredericton, New Brunswick E3B5A3, Canada

[d]Department of Mechanical Engineering, Faculty of Engineering, Universitas Islam Kalimantan MAB, Jl. Adhyaksa No. 2, Banjarmasin 70124, Indonesia

[e]Institut de Recherche en Sciences de la Santé (IRSS-DRCO)/Nanoro, 03 B.P 7192, Ouagadougou 03, Burkina Faso

[f]Gingen technology Co., LTD., Rm. 7, 10F., No.189, Sec. 2, Keelung Rd., Xinyi Dist., Taipei 11054, Taiwan

[g]Department of Chemical Engineering, Ming Chi University of Technology, New Taipei City 24301, Taiwan

[*]Corresponding author (Y.F. Wu): Email: gausswu@mail.mcut.edu.tw

[*]Corresponding author (L.Y. Lin): E-mail: lylin@ntut.edu.tw

Measurements and characterizations

The surface morphology of BVO, NTO and NTO/BVO were examined by field-emission scanning electron microscopy (FE-SEM, Nova NanoSEM 230, FEI, Oregon, USA) and transmission scanning electron microscopy. The compositions and phases of BVO, NTO and NTO/BVO were examined using X-ray diffraction patterns (XRD, X'Pert^3 Powder, PANalytical) and X-ray photoelectron spectroscopy (XPS, VG Scientific ESCALAB 250) measurement with the Al Ka radiation. The light absorbance features of BVO and NTO/BVO was measured via ultraviolet-visible spectroscopy (UV-vis, JASCO V750). The electrochemical performance of BVO, NTO and NTO/BVO photoanodes is measured in a three-electrode system. The Pt wire was acted as the counter electrode and the Ag/AgCl electrode was acted as the reference electrode. The simulated sunlight is the 300-Watt Xe lamp equipped with AM 1.5G filter (Newport). The electrochemical impedance spectroscopy (EIS) was evaluated by potentiostat/galvanostat (PGSTAT 204, Autolab, Eco–Chemie, the Netherlands) equipped with the FRA2 module. The Mott-Schottky equation was shown as follows: $\frac{1}{C^2} = \frac{2(V-V_{fb}-\frac{k_BT}{e})}{\varepsilon\varepsilon_0 A^2 e N_d}$, in which C is differential capacitance, ε is dielectric constant of $BiVO_4$, ε_0 is permittivity of free space, A is area, e is elementary charge, N_d is carrier density, V is applied potential, and V_{fb} is flat band potential, k_B is Boltzmann constant, and T is absolute temperature. The electrolyte contains 0.5 M Na_2SO_4 and 0.5 M Na_2SO_3, which presented the pH value of 9.8. The potentials are calculated versus reversible hydrogen electrode (RHE) via **Equation 1** as follows.

$$E \text{ (vs. RHE)} = E \text{ (}vs.\text{ Ag/AgCl)} + 0.05916 \times 9.8 + 0.197 = E \text{ (}vs.\text{ Ag/AgCl)} + 0.777 \quad (1)$$

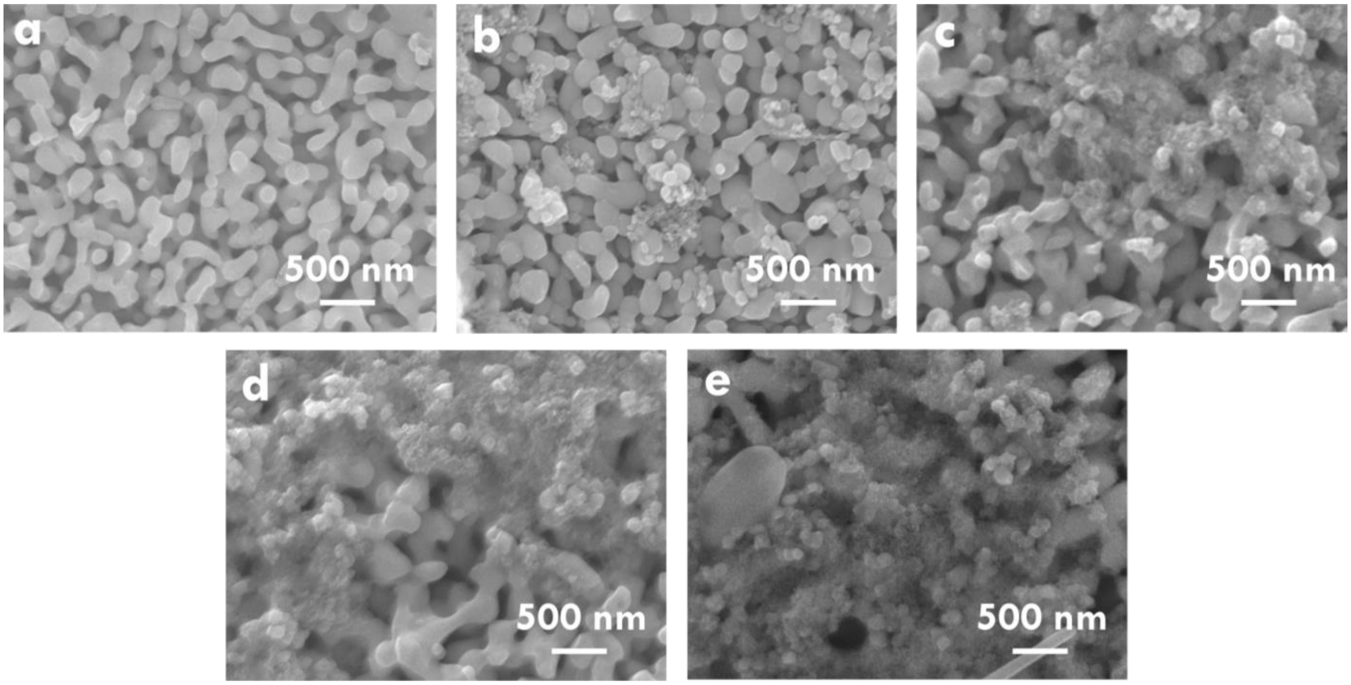

Figure S1 The SEM images of (a) BVO, (b) NTO/BVO-1, (c) NTO/BVO-2, (d) NTO/BVO-3 and (e) NTO/BVO-4 after stability test.
